# Classification of Indonesian adult forensic gender using cephalometric radiography with VGG16 and VGG19: a Preliminary research

**DOI:** 10.2340/aos.v83.40476

**Published:** 2024-05-21

**Authors:** Vitria Wuri Handayani, Ahmad Yudianto, Mieke Sylvia, Riries Rulaningtyas, Muhammad Rasyad Caesarardhi

**Affiliations:** a Doctoral Program of Medical Science, Medical Faculty, Universitas Airlangga, Surabaya, Indonesia; b Nursing Department, Pontianak Polytechnic Health Ministry, Pontianak, Indonesia; c Department of Forensics and Medicolegal, Faculty of Medicine, Universitas Airlangga, Surabaya, Indonesia; d Magister of Forensic Sciences, Postgraduate School, Universitas Airlangga, Surabaya; e Forensic Odontology Department, Dental Medical Faculty, Univesitas Airlangga, Surabaya, Indonesia; f Physics Department, Sains and Technology Faculty, Universitas Airlangga, Surabaya, Indonesia; g Biomedical Department, Sains and Technology Faculty, Universitas Airlangga, Surabaya, Indonesia; h Department of Information Systems, Institut Teknologi Sepuluh Nopember, Surabaya, Indonesia

**Keywords:** cephalometry, gender determination, VGG16, VGG19

## Abstract

**Background:**

The use of cephalometric pictures in dental radiology is widely acknowledged as a dependable technique for determining the gender of an individual. The Visual Geometry Group 16 (VGG16) and Visual Geometry Group 19 (VGG19) algorithms have been proven to be effective in image classification.

**Objectives:**

To acknowledge the importance of comprehending the complex procedures associated with the generation and adjustment of inputs in order to obtain precise outcomes using the VGG16 and VGG19 algorithms.

**Material and Method:**

The current work utilised a dataset including 274 cephalometric radiographic pictures of adult Indonesians’ oral health records to construct a gender classification model using the VGG16 and VGG19 architectures using Python.

**Result:**

The VGG16 model has a gender identification accuracy of 93% for females and 73% for males, resulting in an average accuracy of 89% across both genders. In the context of gender identification, the VGG19 model has been found to achieve an accuracy of 0.95% for females and 0.80% for men, resulting in an overall accuracy of 0.93% when considering both genders.

**Conclusion:**

The application of VGG16 and VGG19 models has played a significant role in identifying gender based on the study of cephalometric radiography. This application has demonstrated the exceptional effectiveness of both models in accurately predicting the gender of Indonesian adults.

## Introduction

According to the Indonesian Disaster Infographics data source, there will be a significant increase in the number of registered disasters in 2022, reaching a total of 3,544 incidents [1]. The 2004 tsunami in Aceh is commonly acknowledged as a highly devastating disaster in terms of human casualties, leading to the untimely death of 165,708 individuals, with a considerable number of them still unidentified [2]. The initial response protocols in Indonesia for managing mass catastrophe victims of unknown identity mostly involve employing visual identification methods, inspecting personal belongings such as jewels, verifying identity cards, and conducting examinations of mobile phone subscriber identity module (SIM) cards. In the event that the victims cannot be reidentified within a few days, a prompt decision will be made to conduct mass burials in order to prevent further deterioration of the remains of over 165,708 individuals, many of whom have become unidentifiable [2]. The expeditious and accurate identification of victims in situations involving a substantial number of individuals requires the utilisation of forensic techniques. This is crucial in order to enhance efficiency, precision, and comprehensiveness in the identification process. The importance of this matter extends beyond humanitarian and emotional concerns for the families impacted, encompassing legal and administrative interests as well [3, 4].

The recognition of gender is a critical aspect of the mass disaster identification process [5]. The application of radiographic techniques for the purpose of determining gender, namely by analysing dental, spinal, and cranial features, can be considered a suitable methodology. One strategy that can be employed in the field of radiology is the utilisation of lateral cephalometry. Cephalometry in radiology is a discipline that encompasses the systematic investigation of quantifying the size and anatomical features of the human cranium. The inception of this field of study can be attributed to Broadbent’s initial exposition in 1931 [6]. Lateral cephalometry provides the advantage of obtaining a thorough visual depiction of the cranial structure and soft tissue contour. Moreover, it facilitates the assessment of numerous anatomical components, including the nasal bones, frontal sinuses, sinus sphenoids, and other pertinent images that contribute to the gender identification process [7].

The integration of software technology has become a fundamental component within the field of forensic odontology. The field of dental and maxillofacial radiography has witnessed significant advancements in artificial intelligence (AI) research, leading to notable breakthroughs in the realm of forensic science [8, 9]. These developments have resulted in the provision of dependable information that aids in decision-making processes. One of the software options being evaluated is a convolutional neural network (CNN). This model resembles the operation of neural networks, specifically through the use of a convoluted layer, and performs operations similar to those conducted by image processing filters [10]. A CNN possesses the capability to effectively recognise images with a level of precision that rivals human performance on a specific dataset, and additionally, it can analyze the characteristics of features that contribute to achieving higher levels of accuracy [11, 12].

The CNN-based Visual Geometry Group (VGG) model has been widely utilised in numerous image-related applications, such as image classification, object detection, and semantic segmentation, due to its ability to enhance performance measures [13, 14]. Notably, the VGG model simplifies the processing by employing a 3 × 3 filter in each layer [10, 13, 14]. The VGG architectures, namely Visual Geometry Group 16 (VGG16) and Visual Geometry Group 19 (VGG19), were developed at Oxford University, with a total of 41 and 47 levels, respectively [13]. The application of similar and smaller filter sizes on VGG16 and VGG19 models is expected to result in the extraction of more complex features while reducing computational demands. This technique holds particular significance in the domain of forensic science, where the precise and efficient execution of gender identification is of utmost importance.

In the current study, there are a number of unanswered concerns regarding the practical viability of computational intelligence within the domain of forensic sciences. Nevertheless, the attainment of generating and altering inputs to yield precise outcomes by computer algorithms remains unrealised. Provided to the context, the objective of this study is to create an additional AI-based model, namely VGG16 and VGG19, to facilitate gender identification.

## Materials and method

A cephalometry image was obtained from the patient’s medical records at the Dental Hospital of Airlangga University in Surabaya. The human subjects ethics board of 316/HERCC.FODM/III/2023 at the Dental Hospital of Airlangga University granted approval for this investigation. This study employed two algorithms. The initial model was the VGG16, followed by the subsequent model, the VGG19. Following that, the sample will be divided into three subsets: 80% of the cephalometry photos will be assigned for training, 10% will be set aside for validation tests, and the remaining 10% will be used for testing. Python is utilised to train, construct, and analyse cephalometric algorithms employing VGG16 and VGG19.

Regarding the inclusion criteria that will be employed, they are as follows:

1. Cephalometric images were obtained from pre-existing cephalometric photographs at the RSGMP FKG Airlangga University in Surabaya.
2. Cephalometric pictures were acquired from individuals of Indonesian descent.
3. Cephalometric photographs are captured using standardised instruments and identical equipment.
4. The cephalometric photograph is in a satisfactory state, with no evidence of superimposition.
5. The cephalometric photograph exhibits a satisfactory state of preservation, devoid of any discernible distortions.
6. Cephalometric photographs are captured by operators possessing a minimum of D3 radiology education, together with a minimum of 1 year of practical experience operating cephalometric equipment.
7. Cephalometric images of individuals between the ages of 18 and 40 years. The cephalometric photograph exhibits a comprehensive dentition in both the mandibular and maxillary regions, with the exception of the third molar.
8. Cephalometric photographs are taken of individuals who either lack orthodontic intervention or possess a prior record of orthodontic intervention.
9. Cephalometric photographs were obtained from individuals lacking a prior record of orthognathic surgery.
10. Cephalometric photographs are taken of individuals who do not possess any documented instances of jaw injuries.

This study employs the following instruments:

1. The cephalometric photographs were captured using equipment that adheres to defined protocols.
2. The cephalometric device employed in this study is the ZULASSUNG THA/HV-GEN Type THA100.
3. Operators of cephalometric equipment possess standardised skills.
4. A computer system equipped with a minimum of two 8-gigabyte random access memory (RAM) modules and a solid-state drive (SSD) with a storage capacity of 1 terabyte.
5. The computer is equipped with the NVIDIA GeForce RTX 3060 graphics processing unit.
6. The utilisation of Python in web development and Google Collaboration

## Research result

This section provides a description of the experimental outcomes. Initially, the criteria for assessing the suggested approach are established. Subsequently, a comparative analysis is conducted between the outcomes of the suggested methodology and those of various contemporary approaches. The present investigation gathered cephalometry radiographs from individuals who sought treatment at Airlangga University Dental Hospital in Surabaya, Indonesia. We choose the suitable 274 dataset format for persons between the ages of 18 and 40 years. Python was used to separate a collection of 274 cephalometric pictures into three groupings. Table 1 demonstrates the allocation of images, with 80% designated for training, 10% for validation, and the remaining 10% for testing.

**Table 1:** Sample frequency distribution.

Classification	Training (80%)	Validating (10%)	Testing (10%)	Total sampling
Male	52	6	7	65
Female	167	21	21	209
Total sampling	219	27	28	274

**Figure 1:**
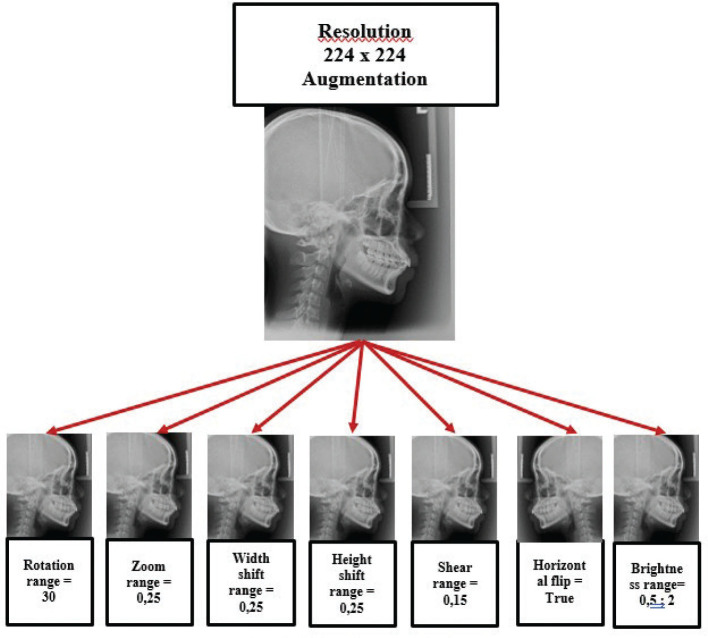
Data set augmentation process.

**Figure 2:**
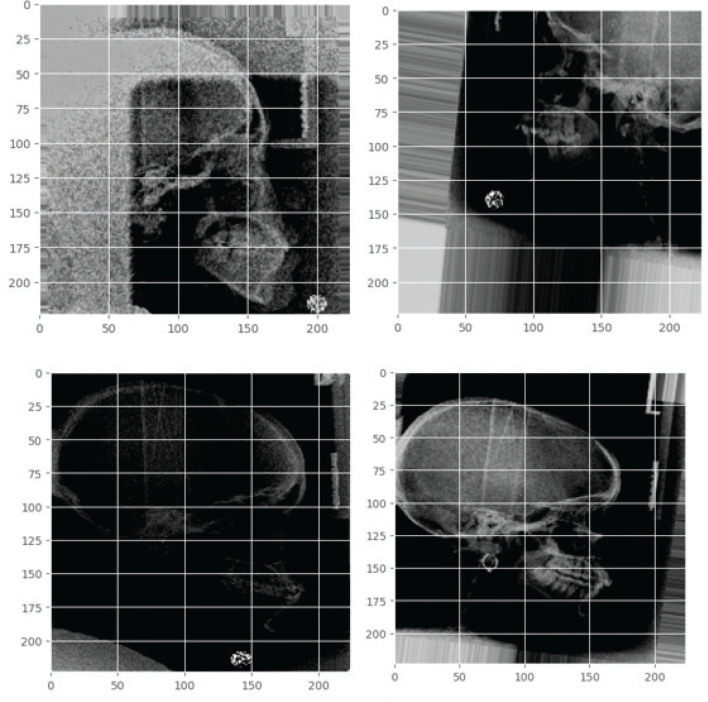
Augmentation feature example.

The traits that were gathered were then chosen manually with the guidance of a radiologist who has been working for 15 years and a dentist who has been practising for 11 years. The aforementioned attributes are thereafter subjected to processing at a resolution of 224 by 224 pixels. Subsequently, a series of augmentation approaches are implemented, encompassing rotation range, zoom range, width shift range, height shift range, shear range, horizontal flip, and change of feature brightness. The incorporation of supplementary images into the training data can potentially address the challenges of overfitting and non-convergence that can manifest in deep learning systems [15]. When the colour of an object has low contrast and brightness compared to the background of a picture, the limited colour information makes it difficult to precisely identify and locate the object within the image. The process is depicted in Figures 1 and 2.

**Figure 3:**
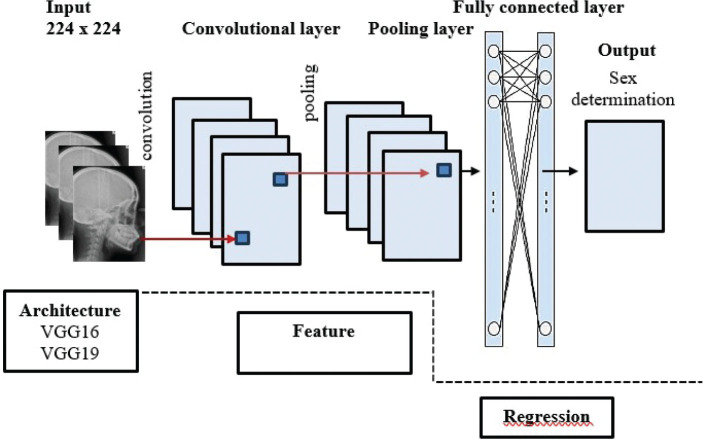
Feature extraction and regression from cephalometric photographs (16).

Machine learning refers to an algorithm that learns from user-generated data in order to predict outcomes. The process of model training continues until the model’s performance reaches its optimal values. The VGG16 and VGG19 algorithm’s development entails the utilisation of classification and regression techniques to construct a gender prediction model, as depicted in Figure 3 [16].

### VGG16 models

VGGNet achieved significant achievement by securing the second position in ImageNet image classification in 2014. Among the several networks inside VGGNet, VGG16 emerged as a particularly high-performing model [17]. The VGG16 model is characterised by its architecture consisting of 16 layers, each storing parameters. These layers are organised into five blocks, with an additional portion of completely connected layers. The VGG16 architecture is a widely utilised pre-trained CNN model that is specifically designed for applications involving image recognition [18].

**Figure 4:**
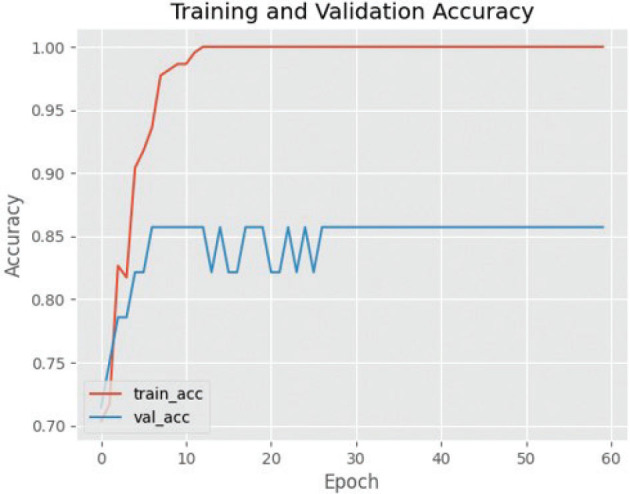
Graph of training and validation success using VGG16. VGG16: Visual Geometry Group 16.

**Figure 5:**
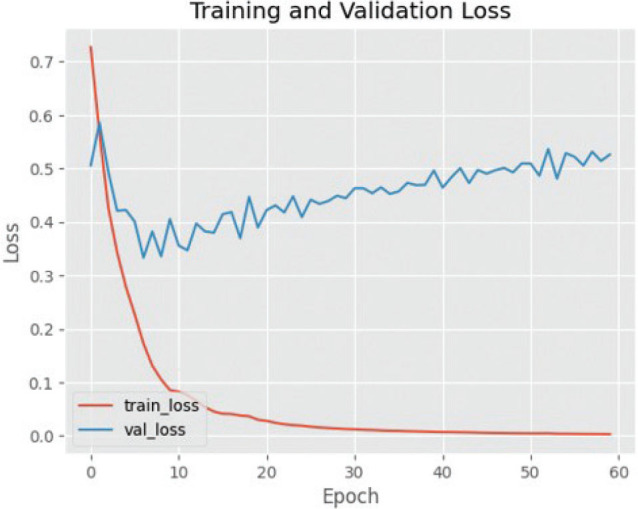
Loss graph in the training and validation process on VGG16. VGG16: Visual Geometry Group 16.

The effectiveness of utilising the VGG16 architecture for training and validating cephalometric images is illustrated by Figures 3, 4 and 5. The efficacy of the observed results is supported by the gradual decrease in loss values illustrated in the training and validation graphs. The training loss and validation loss exhibit a close proximity, with the validation loss marginally surpassing the training loss. In this study, it is observed that the standard deviation of cross-validation accuracies is comparatively higher when compared to the underfit and good-fit models. The training accuracy exhibits a greater value compared to the cross-validation accuracy, indicating a characteristic pattern of an overfit model. However, the magnitude of this difference is large, making it more indicative of the presence of overfitting.

The results of the matrix classification analysis conducted on female and male subjects using the VGG16 model are visually represented in Tables 2 and 3. Tables 2 and 3 present the accuracy and matrix classification results for males and females in the VGG16 model. The findings derived from the use of the VGG16 model to classify gender using a matrix approach, as presented in Table 4, indicate that among the 21 cephalometric variables analysed, about 95% of the predictions made by the VGG16 model align with the female gender. Concurrently, the remaining 5% is indicative of the male gender. On the other hand, the classification of gender in males is associated with a set of six features. In the field of cephalometry, the VGG16 model successfully identified the gender of two cases as female and accurately classified four features as male.

**Table 2:** VGG16 accuracy result.

Classification	f1-score
Male	0.73
Female	0.93
Accuracy result	0.89

VGG16: Visual Geometry Group 16.

**Table 3:** The results of the male and female matrix classification using VGG16 architecture.

*n = 27*	*Predicted: YES*	*Predicted: NO*
*Actual: NO*	TP = 20	TN = 1
*Actual: YES*	FP = 2	FN = 4
	22	5

FP: False positives; TP: true positive; TN: true negative; FN: false negatives.

**Table 4:** VGG19 accuracy result.

Classification	f1-skor
Male	0.80
Female	0.95
Accuracy result	0.93

VGG19: Visual Geometry Group 19.

### VGG19 models

**Figure 6:**
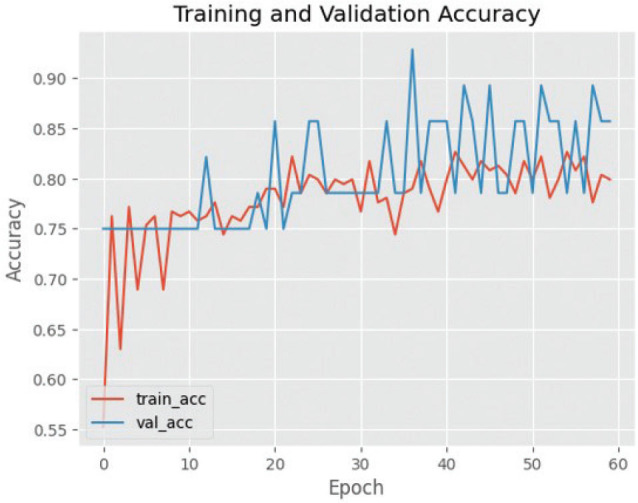
Graph of training and validation success using VGG19. VGG19: Visual Geometry Group 19.

**Figure 7:**
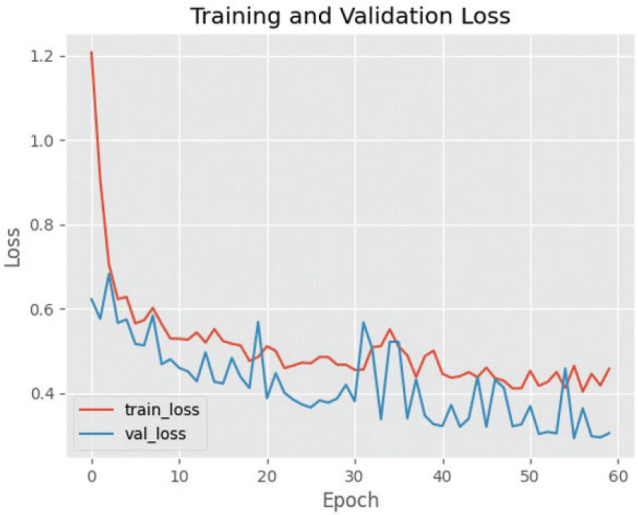
Loss graph in the training and validation process on VGG19. VGG19: Visual Geometry Group 19.

The efficacy of utilising the VGG19 design for training and validating cephalometric images is supported by the convincing data presented in Figures 6 and 7. The aforementioned assertion is reinforced by the visual depiction of the decrease in loss observed during the training and validation phases. While the outcomes of VGG19 differ from those of VGG16, it is worth noting that the standard deviation of cross-validation accuracies is somewhat higher in the former compared to the models exhibiting underfitting and good fit. The training accuracy demonstrates a higher magnitude in comparison to the cross-validation accuracy, suggesting a discernible trend of an overfitting model. Nevertheless, the extent of this disparity is quite small, hence diminishing its significance as an indicator of overfitting.

Tables 4 and 5 show the accuracy measurements and matrix categorisations of VGG19 for male and female individuals. The findings suggest that VGG19 outperforms VGG16, as it achieved 100% accuracy in accurately identifying female cephalometric traits within a sample of 21 instances. On the contrary, the VGG19 model exhibits misclassification by assigning a female label to two instances that possess male cephalometric features. However, it demonstrates appropriate classification by properly identifying four instances as male out of a total of six occurrences. The occurrence under observation can be attributed to disparities in the number of cephalometric samples accessible for males and females, leading to a reduced level of accuracy in the VGG16 and VGG19 models. Table 6 shows the differences of precision, recall, and f1-score values of VGG16 and VGG19.

**Table 5:** The results of the male and female matrix classification using VGG19 architecture.

*n = 27*	*Predicted: YES*	*Predicted: NO*
*Actual: NO*	TP = 21	TN = 0
*Actual: YES*	FP = 2	FN = 4
	23	4

VGG19: Visual Geometry Group 19; TP: true positive; FP: False positives; TN: true negative; FN: false negatives.

**Table 6:** Differences in precision, recall, f1-score values of VGG16 and VGG19.

Algorithm	Variables	Precision	Recall	f1-score	support
VGG16	Male	0.80	0.67	0.73	6
	Female	0.91	0.95	0.93	21
	Accuracy			0.89	27
	Macro average	0.85	0.81	0.83	27
	Weighted average	0.88	0.89	0.89	27
VGG19	Male	1.00	0.67	0.80	6
	Female	0.91	1.00	0.95	21
	Accuracy			0.93	27
	Macro average	0.96	0.83	0.88	27
	Weighted average	0.93	0.93	0.92	27

VGG16: Visual Geometry Group 16; VGG19: Visual Geometry Group 19.

### Comparison of VGG16 and VGG19 results

Precision, accuracy, recall, and precision are important performance indicators employed in categorisation tasks. A true positive (TP) refers to the number of positive samples that have been correctly categorised in the prediction findings. False positives (FP) refer to the number of negative samples that are erroneously identified in the outcome of a prediction. A true negative (TN) refers to the number of positive samples that have been wrongly classified in the prediction results. Finally, false negatives (FN) refer to the number of positive samples that were not identified in the forecast outcomes [19]. The results of accuracy, precision, and recall may be observed in Table 6 of this research.

## Discussion

Estimating a person’s gender becomes the initial identification step, coming before steps for determining their age or ethnicity [20]. The objective of this study is to classify gender identification within the adult population of Indonesia. In their 2017 study, Aurizanti et al. conducted a comparison of craniofacial linear measurements between males and females aged 20–40 years in Indonesia. They used digital lateral cephalometric radiography to measure the craniofacial dimensions [21]. This age range was chosen because it is when the cranial bone stabilises and stops undergoing degenerative changes [21]. Nevertheless, this study employed a sample of individuals between the ages of 18 and 40 years, as we posit that cranial bone development stabilises at 18 years of age.

Considerable advancements have been achieved in the last decade in the application of AI, primarily CNN, in the domain of dentistry, particularly in the field of forensic odontology [22, 23]. Progress in CNNs has been seen in different dental and maxillofacial fields [24]. Oktay, provides that CNN can be effectively used to detect teeth using 100 panoramic dental images with an accuracy of over 90% [25]. Matsuda et al. assessed the efficacy of using CNN technology in Periapical Index (PI) based on ortho-pantomography. They validated the identification accuracy of six distinct CNN designs [26]. Furthermore, the findings indicated that VGG16 proved to be the most effective CNN architecture for PI analysis utilising orthopantomography. In addition, the utilisation of VGG16 pretraining and fine-tuning on the ImageNet dataset resulted in a 100% accuracy rate for the identification [26].

To create an accurate model of CNN algorithm, we require a substantial amount of data, which we refer to as ‘big data’ [27]. The term ‘big data’ refers to large amounts of digital information, including photos and reports, that are stored in electronic formats. The data mentioned above are crucial for the progress of AI in the field of forensic odontology [23, 28]. This study utilised a dataset of 274 lateral cephalometry measurements, comprising 65 males and 209 females. Lateral cephalometry was employed because of its ability to provide anatomical features that may be quantified using lines, angles, or areas [20].

The study provides a comparative analysis of the accuracy, precision, and recall outcomes obtained from VGG16 and VGG19 models. Precision, accuracy, recall, and precision are crucial performance characteristics utilised in categorising jobs to ensure the generation of precise predictions based on the data [29, 30]. Accuracy can be defined as the proportion of correctly identified samples relative to the total number of samples. The evaluation index under consideration has a significant level of intuitiveness, albeit with occasional instances of deception [31]. In situations characterised by an unequal distribution of samples, the accuracy metric tends to demonstrate a bias towards a larger quantity of samples. Precision refers to the proportion of accurately detected and assigned categories in relation to the overall results presented in the reversal results. The recall rate can be defined as the proportion of correctly identified categories that are present in the retrieved results, relative to the overall number of relevant categories [31].

Initially, precision functions as a dependable measure for assessing the outcomes. This can be observed in Tables 3 and 5, where it is evident that the cost associated with FP is quite low. This implies that both VGG16 and VGG19 exhibit a low rate of FP in gender identification, as seen by the findings presented in Table 6. Additionally, accuracy is a quantitative measure that evaluates the frequency with which a machine learning model accurately predicts the desired outcome. Both the VGG16 and VGG19 models exhibit high accuracy, with perfect accuracy being achieved when all predictions made by the model are right. Finally, recall is a quantitative measure that evaluates the frequency at which a machine learning model accurately identifies positive instances. Recall can be computed by dividing the count of genuine positives by the count of positive cases. Tables 3 and 5 demonstrate a notably high TP value, which is further supported by the findings presented in Table 6. This indicates that both the VGG16 and VGG19 models can accurately determine gender through the analysis of cephalometric pictures.

The findings derived from the use of the VGG16 model to classify gender using a matrix approach, as presented in Table 4, indicate that about 95% of the predictions made by the VGG16 model align with the female gender out of the 21 cephalometric variables that were analysed. Concurrently, the remaining 5% of the population is indicative of the male gender. On the other hand, the classification of gender among males is associated with a set of six features. In the field of cephalometry, the VGG16 model successfully identified the gender of two cases as female and accurately classified four features as male. The results of the matrix classification analysis conducted on female and male subjects using the VGG19 model are illustrated in Figure 5. The findings suggest that VGG19 exhibits 100% accuracy in accurately identifying female cephalometric traits within a sample of 21 instances. In contrast, the VGG19 model exhibits misclassification by assigning a female label to two instances that had male cephalometric features. However, it accurately classifies four instances as male out of a total of six occurrences. The observed phenomenon can be attributed to the unequal availability of cephalometric samples for boys and females, resulting in decreased accuracy in the VGG16 and VGG19 models. In other words, there is an imbalance in the data.

Data classification with an imbalanced class distribution has a significant constraint on the effectiveness of conventional classifier learning algorithms [32, 33]. This work highlights the difficulty of implementing data analytics using machine learning techniques, including VGG16 and VGG19 models [33]. According to Kumar, imbalanced data refer to situations when the sample size of one class is much smaller or larger than another class [33]. This study discovered that while the overall accuracy is great, the performance of the male class in the confusion matrix is not satisfactory. The disparity in the sample sizes of males and females has an impact on the performance of prediction models.

The efficacy of the VGG16 and VGG19 models in gender identification has been shown in this study. However, their reliability in accurately recognizing skeletal features corresponding to cephalometric photos in mass disaster scenarios remains dubious. Hence, it is imperative to enhance the instruments and applications grounded in this framework to aid forensic odontologists in devising algorithms that can expedite efficient and prompt identification in scenarios encompassing a substantial number of casualties. While accuracy is a commonly used metric, it can often provide a false representation of performance, particularly when it comes to crucial minority classes. In the present scenario, the minority class being referred to is the male population.

## Conclusion

The VGG16 and VGG19 models have the capability to precisely identify gender by analyzing cephalometric photographs. Nevertheless, the cephalometric model that utilizes the VGG19 architecture in CNN demonstrates superior accuracy in comparison to the VGG16 architecture. The disparity between the female and male samples results in a decline in the performance of this model. Finally, we recommend that future research efforts focus on improving the practicality and precision of the proposed model, such as by refining it to achieve better data balance.
